# Exceptional association of two species of bacteria causing mediastinitis: *Haemophilus influenzae (H. influenzae)* and *Aggregatibacter aphrophilus (A. aphrophilus)*

**DOI:** 10.1186/s12879-018-3269-4

**Published:** 2018-08-16

**Authors:** Badia Belarj, Souhail Dahraoui, Leila Rar, Noureddine Atmani, Mohammed Frikh, Yassine Ben Lahlou, Adil Maleb, Abdelhay Lemnouer, Mahdi Ait Houssa, Abdelatif Boulahya, Mostafa Elouennass

**Affiliations:** 1 Hôpital Militaire d’instruction Mohammed V / Université Mohamed V Rabat, Faculté de médecine et de pharmacie de Rabat / Equipe de recherche ERB/Laboratoire de Bactériologie, Rabat, Morocco; 2 Hôpital Militaire d’instruction Mohammed V / Université Mohamed V Rabat, Faculté de médecine et de pharmacie de Rabat / Service de chirurgie cardiovasculaire, Rabat, Morocco; 3 Centre Hospitalo-Universitaire Mohammed VI Oujda, Université Mohammed Premier Oujda, Faculté de médecine et de pharmacie Oujda / Laboratoire de Bactériologie, Oujda, Morocco

**Keywords:** Post cardiac surgery Mediastinitis, Exceptional association, *Haemophilus influenzae*, *Aggregatibacter aphrophilus*

## Abstract

**Background:**

Post cardiac surgery mediastinitis is the major infectious complication, despite the development of surgical techniques and the application of strict preventive measures.

The *Haemophilus influenzae* mediastinitis is very rare. The mediastinitis caused by the association between *Haemophilus influenzae* and *Aggregatibacter aphrophilus* has never been described to our knowledge.

**Case presentation:**

We report the case of an exceptional combination of *Haemophilus influenzae* and *Aggregatibacter aphrophilus* in a patient operated for single bypass which is complicated by mediastinitis the 10th day after the surgical act.

**Conclusion:**

The conclusion to be drawn from this work is to think in unusual seeds in case of mediastinitis post cardiac surgery for the elaboration of recommendations for antibiotic prophylaxis.

## Background

The postoperative mediastinitis is one of the major infectious complications of cardiac surgery, despite using modern surgical techniques, implementing recommendations regarding antibiotic prophylaxis and conducting a careful skin preparation of the patient.

The incidence of this disease varies between 0.15 to 8% [1].

*Staphylococcus aureus* is the species most frequently isolated in post cardiac surgery, *Haemophilus influenzae* mediastinitis cases are exceptional in addition to the association with *Aggregatibacter aphrophilus* [2].

We report a case of an exceptional combination between HI and AA in a patient with mediastinitis following a single bypass.

## Case presentation

A 74-year old man, hypertensive, diabetic for 30 years, with chronic smoking symptoms, was admitted to the cardiovascular surgery department of the Mohamed V^th^ military teaching Hospital of Rabat, for single bypass. Ten days after surgery, the patient had febrile peaks at 39 °C and purulent sternum discharge. Aerobic and anaerobic blood cultures were performed. The C-reactive protein was at 327 mg per liter and the leukocyte counts to 24*10^3^ cells per microliter, predominantly neutrophils (92%). In immediate postoperative, the patient developed bronchitis with interstitial pictures on chest radiograph but without purulent secretions.

The scanner has objectified the presence of a hypodense mass in the anterior mediastinum.

**Fig. 1:**
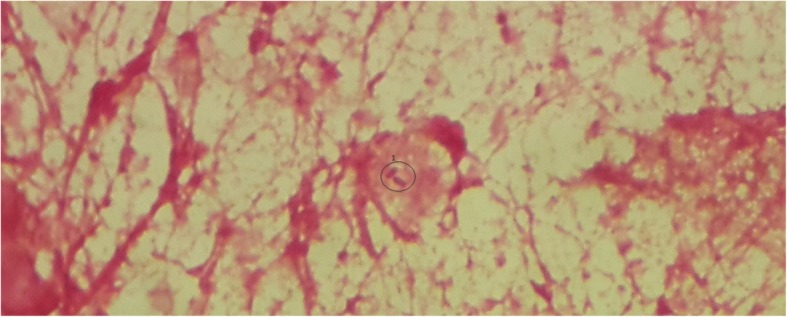
Microscopic picture (Lens * 100) of the pathological sample showing the presence of Gram-negative bacilli; 1:Gram-negative bacilli

Microbiological examination of deep pus drained during a revision surgery of surgical site showed the presence of two bacterial strains (Fig. 1). The identification of bacterial colonies obtained on Chocolate-isovitalex agar, was on biochemical basis using NH API* strips and bacterial grow in presence of the X-factor and the V-factor on Muller-Hinton agar which allowed the isolation of *Haemophilus influenzae* and *Aggregatibacter aphrophilus*. Susceptibility analysis of the isolated strains was carried out with the disc diffusion methodology according to the CA-SFM^*^. It showed the same susceptibility profile for the 2 strains with a susceptibility to the aminopenicillins, cephalosporins third generation, tetracyclines, quinolones and fluoroquinolones, rifampicin and erythromycin, chloramphenicol and imipenem.

**Fig. 2:**
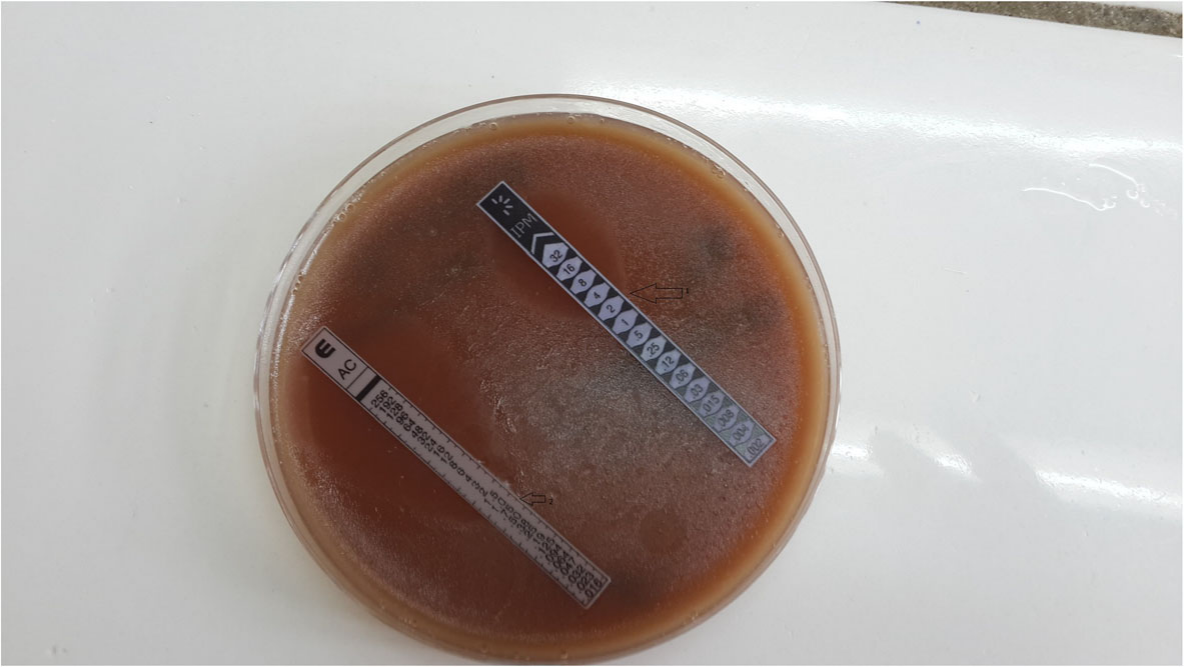
Photographic image of the MIC made by E-test on chocolate agar; 1: MIC imipenem at 2 μg/ml, 2: MIC aminopenicillins at 0.75 μg/ml

The minimum inhibitory concentration (MICs) of aminopenicillins obtained by E-test strip was 0.75 μg/ml and imipenem was 2 μg/ml (Fig. 2).

Blood culture performed during the same episode objectified the presence of the same bacterial strains with the same susceptibility profile to antibiotics.

The patient was treated by empirical antibiotic therapy: ciprofloxacin (800 mg per day), cephalosporins third generation (2 g per day) and vancomycin (2 g per day). Treatment was adjusted to the result of susceptibility studies (Amoxicillin 2 g per day with fluoroquinolone for 3 weeks) with the installation of an irrigation-suction system using suction drains (VAC® therapy).

The evolution marked by the improvement of the patient on the clinical and biological way after 10 days of curative antibiotic therapy.

## Discussion

Postoperative mediastinitis is a deep infection of the sternum which extends beyond the subcutaneous layer. This is one of the most feared complications in cardiac surgery.

Despite improved means of therapeutic treatment, morbidity and mortality remains high with a mortality rate ranging from 11 to 20% [3, 4].

The contamination of the surgical site is often derived from the commensal flora of the human body (patient or health care team) [5].

The most frequently identified organisms are *Staphylococcus aureus* (40–60%) followed by Gram-negative bacilli (BGN) (19% Enterobacteriaceae (*Escherichia coli*, *Proteus species*, *Klebsiella pneumoniae*) and 5 to 10% *Pseudomonas aeruginosa*) and anaerobes. Polymicrobial mediastinitis involving staphylococci, BGN can be seen in 4–30% of cases. *Haemophilus influenzae* mediastinitis cases are rare. The association between *Haemophilus influenzae* and *Aggregatibacter aphrophilus* in mediastinitis is exceptional [1, 2, 6].

In a wide range of mediastinitis in a 8-years study carried out in France (Charbonneau et al.) 3 cases of *Haemophilus influenzae* were found; that being a percentage of 0.9% [7].

In ower knowledge, no associations of *Haemophilus influenzae* with *Aggregatibacter aphrophilus* was found.

Haemophilus is a fastidious and polymorphic Gram-negative Coccobacilli, which usually causes a mild infection on many levels, but may also be responsible for serious infections if it reaches parts of the body where it is unusual to find it.

*H.aphrophilus*, also part of *Haemophilus species*, was recently transferred (2006) in a new genus: Aggregatibacter [8].

Commensal of the oral cavity and upper respiratory tract in children and adults, the two bacteria have been involved in various infections: endocarditis, brain abscess, meningitis, osteomyelitis and arthritis. Mediastinitis remains however a very rare location [9].

The identification of these microorganisms is based on biochemical properties but molecular biology remains more efficient (RNA 16 s or sequencing) [8].

Factors contributing to the development of post-surgical mediastinitis are multiple and dependent on the type of surgery: covers of sternotomy; postoperative bleeding and those dependent on patient history including chronic obstructive pulmonary disease, smoking, diabetes and obesity [1, 2, 5, 6, 8, 10].

In our case, the patient was smoker and diabetic and, he showed an immediate postoperative bronchitis which would likely cause the bacteremia in these two germs.

A study in Nantes on post cardiac surgery mediastinitis, shows the association of this clinical entity with pneumonia in 77% of cases, responsible for a prolongation of mechanical ventilation and, therefore an extension of stay in intensive care. This raises the question of whether the infection was transmitted to the lung by bacterial translocation or bacteremia or conversely if mediastinitis could be secondary to pneumonia [6].

Treatment of mediastinitis includes: the treatment of septic shock and associated visceral failures, antibiotic therapy and aspiration guided by ultrasound or surgical exploration with drainage [2, 5, 11].

As recommended empiric antibiotic therapy should include vancomycin or a cephalosporin, associated with an aminoglycoside or a fluoroquinolone with adjustment to the results of susceptibility testing for a period of 3 weeks [1].

The beta-lactams are top choice antibiotics for treating infections with *H.influenzae* both in adults and in children. However, there are many mechanisms of resistance, the most common being the production of beta-lactamase. It is primarily TEM-type enzyme. A decreased susceptibility to beta-lactams by modification of the penicillin binding proteins (PBPs) target this antibiotic family is another mechanism which can add an efflux mechanism [7, 8, 10, 12, 13].

The in vitro activity of various antibiotics tested showed that cefotaxime and cefpodoxime have better activity in terms of MIC 50 and MIC interval, followed by amoxicillin + clavulanic acid that restores the activity of amoxicillin over the stem producing only beta-lactamase. Cefuroxime and cefaclor are less active in vitro regardless of the phenotype of the strains tested. Similar results have been reported in Europe and North America [7, 8, 10, 12, 13]. The Poor dissemination of beta-lactams and glycopeptides bone level often leads to associate with other molecules, such as fluoroquinolones, rifampin or fucidin [14].

For our patient, antibiotic therapy was at first probabilistic based on vancomycin associated with fluoroquinolone then was switched to aminopenicillin following the result of MICs with fluoroquinolone for a period of 3 weeks.

## Conclusion

The *Haemophilus influenzae* mediastinitis are very rare while the association with *A.aphrophilus* is exceptional whose inoculation mechanism remains undocumented in our case.

Knowing that *H.influenzae* culture requires the presence of V and X factors that are present in fresh blood, it is likely that the bleeding occurred postoperatively in our patient was a favorable condition for the growth and multiplication of the germ.

## Abbreviations

*A. aphrophilus*: *Aggregatibacter aphrophilus*
BGN: Gram-negative bacilli
CA-SFM*: Antibiogram committee of the French Society for Microbiology
*H. influenzae*: *Haemophilus influenzae*
MIC: Minimum inhibitory concentration
NH API*: System for the identification of most Neisseria and Haemophilus spp.
PBPs: Penicillin binding proteins
RNA 16 s: Ribo nucleic acid 16 s
